# Maca (*Lepidium meyenii*) as a Functional Food and Dietary Supplement: A Review on Analytical Studies

**DOI:** 10.3390/foods15020306

**Published:** 2026-01-14

**Authors:** Andreas Wasilewicz, Ulrike Grienke

**Affiliations:** Division of Pharmacognosy, Department of Pharmaceutical Sciences, Faculty of Life Sciences, University of Vienna, Josef-Holaubek-Platz 2, 1090 Vienna, Austria

**Keywords:** *Lepidium meyenii*, maca, Brassicaceae, LC-MS, analysis, quality control

## Abstract

Maca (*Lepidium meyenii* Walp.), a Brassicaceae species native to the high Andes of Peru, has gained global attention as a functional food and herbal medicinal product due to its endocrine-modulating, fertility-enhancing, and neuroprotective properties. Although numerous studies have addressed its biological effects, a systematic and up-to-date summary of its chemical constituents and analytical methodologies is lacking. This review aims to provide a critical overview of the chemical constituents of *L. meyenii* and to evaluate analytical studies published between 2000 and 2025, focusing on recent advances in extraction strategies and qualitative and quantitative analytical techniques for quality control. Major compound classes include macamides, macaenes, glucosinolates, and alkaloids, each contributing to maca’s multifaceted activity. Ultra-(high-)performance liquid chromatography (U(H)PLC), often coupled with ultraviolet, diode array, or mass spectrometric detection, is the primary and most robust analytical platform due to its sensitivity, selectivity, and throughput, while ultrasound-assisted extraction improves efficiency and reproducibility. Emerging techniques such as metabolomics and chemometric approaches enhance quality control by enabling holistic, multivariate assessment of complex systems and early detection of variations not captured by traditional univariate methods. As such, they provide complementary, predictive, and more representative insights into maca’s phytochemical complexity. The novelty of this review lies in its integration of conventional targeted analysis with emerging approaches, comprehensive comparison of analytical workflows, and critical discussion of variability related to phenotype, geographic origin, and post-harvest processing. By emphasizing analytical standardization and quality assessment rather than biological activity alone, this review provides a framework for quality control, authentication, and safety evaluation of *L. meyenii* as a functional food and dietary supplement.

## 1. Introduction

Maca (*Lepidium meyenii* Walp.), also known as Peruvian ginseng [1], is a herbaceous perennial plant native to the high-altitude regions of the central Andes in Peru, particularly at elevations of 3800 to 4500 m [2]. Belonging to the Brassicaceae family, maca is one of the few cultivated crops capable of thriving in extreme alpine climates, characterized by intense sunlight, low temperatures, and poor, rocky soils. The plant forms a rosette of frilly leaves and develops a fleshy, hypocotyl-root structure that resembles a small turnip, which is the primary part used for both nutritional and medicinal purposes [3].

Dried hypocotyls of maca have been consumed for over two millennia by indigenous Andean populations [1,4]. Traditionally, the roots are sun-dried and either boiled to prepare a porridge-like drink or ground into flour for breads and cakes [2]. Maca has long been used in Andean medicine as an adaptogen and tonic to improve the reproductive function in both men and women, reduce stress, prevent estrogen-deficient bone loss, and for its claimed anticarcinogenic activity [1]. Its consumption was also believed to fortify immune function and support resilience facing harsh environmental conditions [2].

Maca is increasingly recognized as a functional food, defined as food that provides health-promoting effects beyond basic nutrition [5,6]. Functional food encompasses a broad range of food types, including probiotic and prebiotic foods, plant foods rich in secondary metabolites, omega-3–rich foods, fortified products, and traditional herbal foods [5]. Probiotic foods, such as yogurt and fermented vegetables, modulate gut microbiota and improve digestive and immune health, while prebiotic fibers selectively stimulate beneficial microbes [7]. Plant-based foods rich in polyphenols, flavonoids, and glucosinolate-derived compounds (e.g., berries, green tea, and cruciferous vegetables) have been associated with antioxidant, anti-inflammatory, and metabolic benefits [8]. Omega-3–rich foods, particularly fatty fish, provide cardiovascular and neurological health benefits [9]. Within this context, traditional herbal foods like maca represent a distinct subgroup characterized by historical use and unique phytochemicals, though clinical evidence remains comparatively limited. Placing maca within this broader functional food context, highlights both its similarities to other bioactive-rich plant foods and the need for further rigorous clinical research to substantiate its health claims.

**Figure 1:**
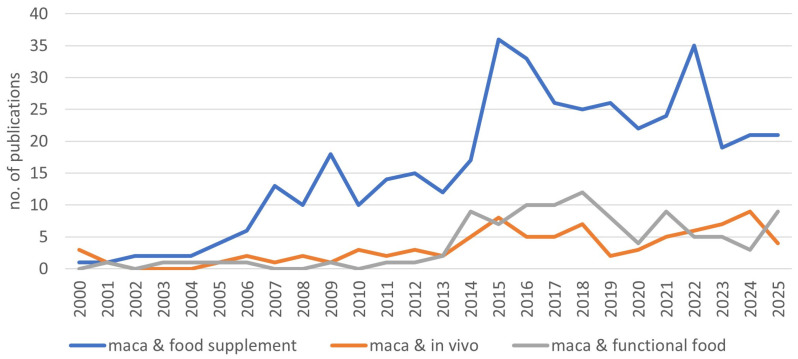
Number of SciFinder-indexed publications on maca using the key words “food supple-ment”, “functional food”, and “in vivo”.

The pharmacological and nutritional properties of maca have been increasingly investigated over the past decade [1]. This is illustrated in Figure 1 which shows the number of SciFinder-indexed publications on maca using the key words “food supplement”, “functional food” and “in vivo”. Due to its use as natural tonic against fatigue and stress, and as a fertility enhancer, maca has been increasingly offered as functional food or food supplements on the US and European markets since the 1990s [10]. Potential pharmacological and toxicological properties of maca’s hypocotlys have recently been reviewed by Ulloa del Carpio et al. [11]. Bioactivity is largely attributed to maca’s rich and diverse secondary metabolite profile, including unique compounds such as fatty acid derivatives (macamides and macaenes), sulfur-containing compounds (glucosinolates and thiohydantoins), alkaloids, lignans, flavonoids, and sterols [12].

Despite growing interest, accurately profiling maca’s chemical composition remains challenging. Composition and concentration of key bioactive compounds vary depending on ecotype (yellow, red, or black hypocotyls) [13,14,15], cultivation location, altitude, harvest time, and processing method [1,2,16]. The chemical complexity of maca, including polar and non-polar constituents, unstable glucosinolates, and minor compounds, makes extraction, separation, and detection technically demanding. Traditional analytical approaches often target only a subset of compounds, risking incomplete characterization. This variability complicates quality control, product standardization, and comparative pharmacological studies.

Over the past two decades, a wide range of analytical strategies have been developed, including high-performance liquid chromatography (HPLC), gas chromatography-mass spectrometry (GC-MS), ultra-performance liquid chromatography (UPLC), liquid chromatography-tandem mass spectrometry (LC-MS/MS), and nuclear magnetic resonance (NMR) spectroscopy [17]. These methods provide valuable qualitative and quantitative data. However, systematic assessments of sensitivity, selectivity, and throughput for maca are limited. Moreover, chemometric and multivariate approaches are not yet fully integrated into standard workflows, which restricts holistic interpretation of complex datasets and reproducibility across laboratories. Emerging techniques such as metabolomics as well as chemometric approaches enhance quality control by enabling holistic, multivariate assessment of complex systems and the early detection of variations not captured by traditional univariate methods, e.g., to authenticate botanical origin. As such, they provide complementary, predictive, and more representative insights into maca’s phytochemical complexity.

Despite growing interest and several recent reviews on maca, these works have largely focused on its pharmacological effects or general phytochemistry. The novelty of this review lies in its focus on analytical methodologies, providing a critical, comparative evaluation of extraction, separation, detection, and validation strategies. By integrating conventional targeted analyses with emerging metabolomics and chemometric approaches, this review addresses key gaps in current research, including variability due to phenotype, geographic origin, and post-harvest processing. By emphasizing analytical standardization and holistic quality assessment rather than biological activity alone, this review establishes a framework for improving quality control, authentication, and safety of maca-based functional foods and dietary supplements.

## 2. Chemical Constituents

The dried hypocotyls of *L. meyenii* predominantly contain hydrolysable carbohydrates (59.0%) followed by proteins (10.2%) and lipids (2.2%), showing similar composition of primary metabolites as carrots (*Daucus carota*) [18].

**Figure 2:**
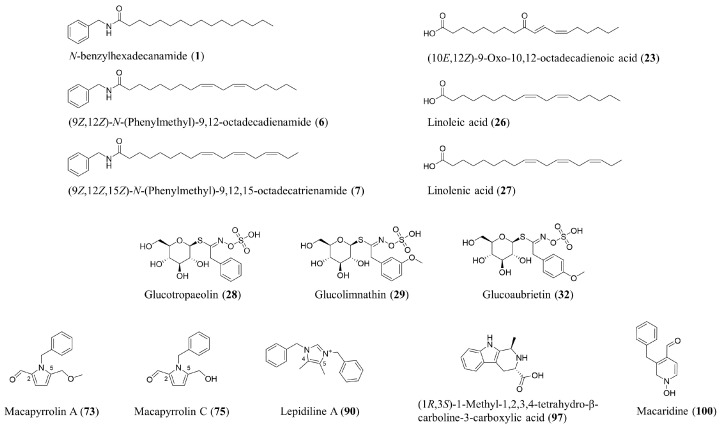
Chemical structures of selected secondary metabolites from maca (*L. meyenii*).

Additionally, the chemical profile of *L. meyenii* hypocotyls encompasses a broad range of secondary metabolites that are in the focus of this review, including fatty acid derivatives (macamides and macaenes), sulfur-containing compounds (glucosinolates and thiohydantoins) and alkaloids (Figure 2). In total, over 100 secondary metabolites were unambiguously identified in maca hypocotyls. Table S1 shows reported secondary metabolites which were identified either (i) by isolation combined with rigorous structure elucidation (e.g., by NMR) or (ii) by comparison with authentic standards. Otherwise, compounds that were annotated solely based on LC-MS without further structure identification were not included.

### 2.1. Macamides and Macaenes

The most investigated compound class of maca are its macamides (**1**–**21**) (Figure S1), also denoted as alkamides that are composed of unbranched and even-numbered fatty acids connected by an amide bond to benzylamine moieties. The majority of the known macamides (**4**–**13**) consists of a benzylamine attached to a fatty acid consisting of 18 carbon atoms with different degrees of unsaturation (1 to 3 double bonds, (*E*) and (*Z*) configuration) and oxidation (maximum of one keto group) [19,20,21,22,23]. In contrast, the most abundant macamide (**1**) is composed of benzylamine and palmitic acid, a saturated fatty acid with 16 carbon atoms [21]. Moreover, additional macamides with methoxylated benzylamine moieties (**14**–**19**) and an alkyl chain length of 24 carbon atoms (**20**, **21**) were described [22,24,25]. Another closely related compound class are the macaenes (Figure S2) which are unbranched, even-numbered, unsaturated, and oxidized fatty acids. To date, only a few macaenes (**22**–**25**) were reported for *L. meyenii* hypocotyls that are all based on a fatty acid with an 18-carbon chain (i.e., stearic acid) [21,23,26]. Moreover, the occurrence of the unsaturated fatty acids (**26**, **27**) in maca’s hypocotyls is routinely reported in the literature [27].

While macamides have been the primary focus of maca research due to their relatively high abundance and well-documented pharmacological activities such as neuroprotective, anti-fatigue, and endocrine-modulating effects [28], macaenes represent a less-studied but potentially important class of bioactive compounds. Structurally, macaenes may serve as biosynthetic precursors to macamides and could contribute synergistically to the overall biological effects of maca [29]. Despite their lower concentrations, macaenes may play a role in modulating or enhancing the activity of macamides, a possibility that remains largely unexplored. From an analytical perspective, macamides are easier to detect and quantify due to their distinctive amide moieties, whereas the lipophilic nature of macaenes makes their extraction and measurement more challenging [23,30]. Consequently, quality control studies and pharmacological evaluations that focus solely on macamides may underestimate the total bioactive potential of maca. A more integrative approach that considers both macamides and macaenes is therefore essential for accurately characterizing maca’s chemical profile and for understanding the full spectrum of its biological effects.

### 2.2. Sulfur-Containing Compounds: Glucosinolates, Thiohydantoins, and Related Compounds

Several glucosinolates (Figure S3) are described for *L. meyenii*, including aromatic (**28**–**32**) [26,31,32,33], aliphatic (**33**–**35**) [13,31] and indole (**36**–**38**) [31] congeners. Early studies on maca reported glucoaubrietin (**32**) as a major glucosinolate [33]. However, more recent analyses from the year 2011 indicate that this was likely a misidentification of its regioisomer, glucolimnanthin (**29**), which together with glucotropaeolin (**28**) constitutes the predominant glucosinolates in maca [31]. This correction has important implications for quantitation studies prior to 2011, as unresolved structural isomers may have led to inaccurate reporting of glucosinolate profiles and concentrations. Accurate characterization therefore requires high-resolution chromatographic separation and spectral confirmation to distinguish glucolimnanthin (**29**) from glucoaubrietin (**32**), ensuring reliable assessment of maca’s chemical composition.

Another sulfur-containing compound class of maca are the thiohydantoins (**39**–**68**) with diverse chemical scaffolds (Figure S4) [34,35,36,37,38,39,40]. Most thiohydatoins (**39**–**58**) reported possess one stereo center, for which both enantiomers were found (except of **53**). While the presence of both enantiomers is acknowledged, the analytical challenges associated with chiral separation are rarely discussed in the literature dealing with the analysis of maca constituents. Chiral HPLC with appropriate stationary phases or circular dichroism coupled to chromatographic methods would be required to differentiate and quantify enantiomers effectively, but such techniques have not been systematically applied in maca studies despite their demonstrated utility in resolving thiohydantoin stereoisomers in other contexts [41]. Consequently, there is a gap in current analytical workflows for maca that limits accurate characterization of thiohydantoin enantiomer distributions and may obscure relationships between stereochemistry and bioactivity.

Moreover, several structurally related compounds, including two hydantoins (**69**, **70**), one urea derivative (**71**) and one thionamide (**72**) were reported for maca hypocotyls [42].

### 2.3. Alkaloids

A diverse set of alkaloids are reported for maca, including pyrrole alkaloids and imidazole alkaloids as the most prominent classes (Figure S5). Most pyrrol alkaloids (**73**–**81**) are based on the same scaffold with a benzyl moiety attached to the pyrrol’s nitrogen atom, a formaldehyde residue in position C-2 and a methylhydroxy group in position C-5 [43,44,45,46]. The structures are further substituted, e.g., by hydroxylation, methylation or methoxylation, leading to a series of structural congeners. In recent years, truncated (**82**, **83**) and dimeric (**84**, **85**) pyrrol alkaloids were isolated from *L. meyenii* hypocotyls [46]. In addition, a couple of pyrrolidine alkaloids (**86**–**89**) were identified with a similar scaffold as the pyrrol alkaloids but with an acetone moiety in position C-2 and a keto group in position C-5 [45]. All known imidazole alkaloids (**90**–**96**) of maca are quaternary alkaloids which are methylated in positions C-4 and C-5 and contain at least one benzyl moiety attached to an imidazole nitrogen atom [44,47,48]. Three β-carboline alkaloids (**97**–**99**) are known for maca, which might be potentially harmful for human health as other structurally related β-carbolines have shown mutagenic, neurotoxic and carcinogenic properties [3,44]. Nevertheless, compound **97** was found in several fruits and is considered as non-toxic when consumed in complex matrices [49,50]. So far, no toxic effects were associated with β-carbolines after the consumption of maca-containing products. One of the first reported alkaloids for *L. meyenii* was the pyridine alkaloid macaridine (**100**) [21]. Its structure was recently revised to the pyrrol alkaloid macapyrrolin C (**75**) [44]. Lately, two amidine alkaloids (**101**, **102**) were isolated from maca and were reported as new natural compounds [44].

### 2.4. Miscellaneous Compounds

Additional constituents were reported for *L. meyenii* hypocotyls, including polysaccharides [51], lignans [52], flavonoids [53] and sterols [30], highlighting the chemical diversity of maca.

## 3. Analytical Techniques

### 3.1. Qualitative Analysis

With more than 100 known secondary metabolites, the identification and isolation of new natural products from *L. meyenii* remains a challenging task. In recent years, several studies using UHPLC-MS have been reported for qualitative analysis and tentative identification of constituents in maca extracts. To perform a comprehensive chemical profiling of maca, Zhou et al. applied an UHPLC coupled to high resolution orbitrap mass spectrometry. Using a C_18_ column and a gradient of acidified water and acetonitrile, more than 160 constituents were detected, including macamides, glucosinolates and various alkaloids [54]. The study of Xia et al. focused on the analysis of macamides and macaenes using an UHPLC-QTOF-MS. The separation was achieved by a C_18_ column, using acidified water and acetonitrile as mobile phase. In total, 35 macamides and macaenes were annotated in air-dried maca samples [55]. Interestingly, macaenes were detected only in negative ion mode, whereas macamides were identified in both ion modes, with stronger ionization observed in positive mode.

To gain insights into the chemical diversity of imidazole alkaloids in maca, Le et al. have recently applied feature-based molecular networking. Hereby, UHPLC-QTOF-MS and MS/MS analyses were performed in positive ion mode. In the resulting molecular network generated, seven known imidazole alkaloids were identified, while amidine alkaloids were identified for the first time in maca [44]. Given the efficient ionization of maca constituents, molecular networking may represent a valuable and timesaving strategy for the identification and targeted isolation of new constituents in future research. However, limitations include the reliance on spectral libraries and in silico predictions, which can reduce annotation confidence, especially for novel compounds without reference spectra. Experimental validation is therefore still required to confirm the identities of newly suggested molecules. Thus, to elucidate the structure of a new compound, comprehensive NMR experiments are required, which may be complemented by additional techniques (e.g., polarimeter, circular dichroism) to establish the absolute configuration. Alternatively, compounds can be identified by comparison with authentic standards, a strategy already employed for the identification of macamides from *L. meyenii* [55].

### 3.2. Quantitative Analysis

Several quantitation studies (I–XVII) were performed that shed light on the chemical composition of *L. meyenii* hypocotyls (Table 1). For extract preparation, dried and powdered maca hypocotyls are typically used. Most studies employ methanol or aqueous methanol as the extraction solvent, with sonication frequently used as the extraction method. HPLC-UV is the most widely utilized analytical technique, primarily for the quantitation of macamides and glucosinolates. In one of the first quantitative studies (I) in 2002, Ganzera et al. employed a C_12_ column, reporting superior resolution and peak symmetry compared C_8_ or C_18_ columns. Subsequent HPLC-based quantitation studies, however, predominantly used C_18_ columns [27]. Mobile phases typically consist of water and acetonitrile, sometimes with additives such as trifluoracetic acid at varying concentrations. Interestingly, Xu et al. (XIII-A) selected 10 mM aqueous ammonium phosphate as hydrophilic component of the mobile phase, achieving better resolution than with other aqueous solutions [30]. With the exception of the studies by Fu et al. [29] (XIV) and Chen et al. [56] (VI), all quantitative analyses employed gradient elution. The composition of the mobile phase generally depends on the compound class being analyzed, with more polar phases applied for glucosinolates and more lipophilic phases for macamides. Additionally, different detection wavelengths are generally applied for these compound classes: macamides were quantified at 210 and 280 nm, while glucosinolates are usually detected between 227 and 235 nm. Interestingly, only one study reports (XIII-A) the simultaneous quantitation of macamides and glucosinolates using UV at 210 nm as detection method [30]. In this study, a Waters UHPLC system was used, while chromatography was performed on a Waters Acquity UHPLC HSST3 column (100 mm × 2.1 mm, 1.8 µm; 35 °C) applying gradient elution with 10 mM aqueous ammonium phosphate and acetonitrile as mobile phases (flow rate of 0.3 mL/min). With this method, eight macamides and two glucosinolates could be quantified within 15 min, showcasing an effective and rapid analysis of maca products. More recent studies increasingly use UHPLC, resulting in shorter run times—as exemplified in the study presented above—and MS, enabling the quantitation of all three major compound classes, i.e., macamides, glucosinolates and alkaloids. ESI has yet been the only ionization technique used, while time-of-flight mass spectrometer and triple quadrupole mass spectrometers have been applied for the quantitation of maca samples. Recently, Le et al. [3] (XVII) have reported an UHPLC-QqQ-MS^2^ method for the quantitation of imidazole, pyrrole and β-carboline alkaloids in maca samples. For the chromatographic separation, a Waters Acquity UHPLC system was used, endowed with an Acquity BEH C_18_ column (100 mm × 2.1 mm, 1.7 µm; 40 °C), which was selected due to its ubiquity. A 12 min method was developed, using gradient elution with water and acetonitrile both containing 0.1% formic acid and a flow rate of 0.5 mL/min. The UHPLC system was coupled to a triple quadrupole equipped with an ESI source, operated in the multiple-reaction monitoring mode for quantification. Cone and collision voltages were individually determined for each standard in the positive ion mode. The remaining parameters, including capillary voltage (3.5 kV), RF lens (0.1 V) and desolvation gas flow (900 L/h) were determined by analyzing an internal and secondary standard mixture, revealing source temperature (set at 140 °C) and desolvation temperature (set at 500 °C) as decisive parameters. Although two imidazole alkaloids could not be separated by using formic acid as additive, the researchers still opted for formic acid due to its routine analytical application and its enhancing effect on positive ionization. Additionally, a complete chromatographic separation is not required when using the MRM mode. This developed method was used for the quantitation of eleven alkaloids in maca food supplements, providing a sensitive and comprehensive analysis for the quality control of maca alkaloids in commercial products.

Isothiocyanates, which are degradation products of glucosinolates, can be quantified by GC-MS since they are volatile compounds without any derivatization required [26,30]. In addition, in one study (X), qNMR was applied for the quantitation of the total macamide content of maca samples [57]. A 600 MHz Bruker AVIII spectrometer with cryogenic probe was used to record ^1^H NMR spectra of the samples dissolved in CDCl_3_. For the quantitative analysis, the characteristic proton signal at *δ*_H_ 4.30–4.50 (d, *J* = 6.0 Hz) of the benzylamine methylene was exploited as key quantitative signal [57]. This study enabled a rapid comparison of the total macamide content of maca hypocotyls of different color and origin.

**Table 1:** Reported sample preparation techniques and quantitation methods applied to maca constituents (in chronological order starting with the oldest study).

Study No.	Sample	Extraction Solvent	Extraction Method	Quantitation Method	Conditions	Investigated Compounds	Ref.
I-A	Dried and powdered hypocotyl	70% Aqueous ethanol	n. d.	HPLC-UV ^1^ (235 nm)	Bondapak C_18_, H_2_O + 0.1% TEA/MeOH, gradient, 70 min, 4 mL/min, ^2^	Glucosinolates (**28**, **29**, **33**, **38**)	[26]
I-B		*n*-Hexane	n. d.	GC-MS ^1^ (QqQ)	DB-5 column, helium, gradient, 57 min, 1 mL/min	Isothiocyanates	
II	Dried hypocotyl, dietary supplements	Methanol	Sonication (10 min)	HPLC-UV (210, 280 nm)	Synergi Max-RP (C_12_), H_2_O + 0.025 TFA/ACN + 0.025% TFA, gradient, 35 min, 40 °C, 1.0 mL/min	Macamides (**1**, **8**), macaene (**22**), fatty acids (**26**, **27**)	[27]
III	Dried and powdered hypocotyl, dietary supplements	Petroleum ether	Shaking (24 h, 150 rpm)	HPLC-UV (210 nm)	Zorbax XDB C_18_, H_2_O + 0.005 TFA/ACN + 0.005% TFA, gradient, 30 min, 40 °C, 0.8 mL/min	Macamide (**1**)	[19]
IV	Dried and powdered red hypocotyls	Water	Decoction (60 min)	HPLC-UV (230 nm)	C_18_ column, H_2_O/20% aqueous ACN, gradient, 31 min, 1.5 mL/min, ^2^	Glucosinolate (**28**)	[58]
V	Dried and powdered hypocotyls of different color	Methanol	Sonication (20 min)	HPLC-UV (210, 280 nm)	Synergi Max-RP (C_12_), H_2_O + 0.025 TFA/ACN + 0.025% TFA, gradient, 60 min, 40 °C, 1.0 mL/min	Macamides (**1**, **8**, **9**), macaene (**22**)	[13]
VI	Dried and powdered hypocotyls of different color and origin	Petroleum ether	Ultrasonication	HPLC-UV (210 nm)	Zorbax XDB C_18_, H_2_O/ACN, gradient, 30 min, 40 °C, 0.8 mL/min	Macamides (**1**–**7**, **14**–**17**), fatty acids (**26**, **27**)	[56]
VII	Powdered hypocotyls of different origin	Methanol	Ultrasonication (30 min)	UHPLC-MS (ESI, QqQ)	Thermo Hypersil-Gold C_18_, H_2_O + 0.2% FA/ACN, gradient, 15 min, 30 °C, 0.3 mL/min	Macamides (**1**, **14**), glucosinolates (**28**–**30**), alkaloid (**75**)	[54]
VIII	Dried and powdered hypocotyls	Petroleum ether	Ultrasonication (50 °C, 15 min)	HPLC-MS (QTOF)	XTerra C_18_, H_2_O/ACN/FA, isocratic, 35 min, 30 °C, 0.6 mL/min	Macamides (**1**, **4**–**7**, **15**–**16**)	[59]
IX	Dried and powdered hypocotyls	Methanol	Ultrasonication (40 °C, 60 min)	HPLC-UV (210, 280 nm)	Zorbax XBD-C_18_, H_2_O/ACN + 0.005% TFA, gradient, 45 min, 40 °C, 1 mL/min	Macamides (**1**, **6**, **7**, **11**, **12**), macaenes (**23**, **24**)	[23]
X	Dried and powdered hypocotyls	Ethyl acetate/methanol (2:1)	Ultrasonication (60 min), column chromatography	^1^H qNMR	600 MHz, cryoprobe, CDCl_3_	Total macamides	[57]
XI	Hypocotyls dried in different conditions	Methanol	Ultrasonication (40 °C, 60 min)	HPLC-UV (210, 280 nm)	Zorbax XDB C_18_, H_2_O/ACN + 0.005% TFA, gradient, 45 min, 40 °C, 1 mL/min	Macamides (**1**, **6**, **7**, **11**, **12**), macaenes (**23**–**25**)	[55]
XII	Dietary supplements	70% Aqueous methanol	Sonication (10 min)	HPLC-UV (227 nm)	Luna C_18_(2), H_2_O + 0.1% TFA/ACN + 0.1% TFA, gradient, 30 min, 23 °C, 1.0 mL/min	Glucosinolates (**28**–**31**)	[32]
XIII-A	Dried and powdered hypocotyls	75% Aqueous methanol	Ultrasonication (30 min)	UHPLC-UV (210 nm)	Acquity HSST3 column (C_18_), 10 mM aqueous (NH_4_)_3_PO_3_/ACN, gradient, 14 min, 35 °C, 0.3 mL/min	Macamides (**1**, **3**, **5**–**7**, **14**, **16**, **17**), glucosinolates (**28**, **29**)	[30]
XIII-B		Ethyl acetate	Ultrasonication (60 min)	GC-MS (EI)	SH-Rxi-1 MS column, helium, gradient, 27 min, 1 mL/min	Isothiocyanates	
XIV	Powdered hypocotyls	Methanol	n. d.	UHPLC-MS (ESI, TOF)	Acclaim^TM^ RSLC 120C_18_, H_2_O/75% aqueous ACN + 0.05% FA, isocratic, 9 min, 35 °C, 0.4 mL/min	Macamides (**1**, **5**–**7**, **16**), fatty acids (**26**, **27**)	[29]
XV	Dried hypocotyls	50% Aqueous ethanol	Ultrasonication (3 × 30 min)	HPLC-MS (ESI-QTOF)	Zorbax Eclipse Puls RP-18, H_2_O + 0.1% FA/ACN + 0.1% FA, gradient, 45 min, 0.2 mL/min, ^2^	Glucosinolate (**28**)	[60]
XVI	Dried and powdered hypocotyls	Deep eutectic solvents	Ultrasonication (40 °C, 30 min)	HPLC-UV (210 nm)	Zorbax XDB C_18_, H_2_O/ACN, gradient, 30 min, 40 °C, 0.8 mL/min	Macamides (**1**, **6**, **7**, **16**, **17**)	[61]
XVII	Powdered hypocotyls, dietary supplements	75% Methanol	Sonication (30 min)	UHPLC-MS (ESI, QqQ)	Acquity BEH C_18_, H_2_O + 0.1% FA/ACN + 0.1% FA, gradient, 10 min, 40 °C, 0.5 mL/min	Alkaloids (**73**, **75**, **80**, **90**–**94**, **97**, **98**)	[3]

^1^, semi-quantification; ^2^, column temperature not provided; n. d., not described.

Most quantitation studies have been properly validated, assessing parameters such as accuracy, precision, range of linearity, detection limit, and quantitation limit. Table 2 provides an overview of sensitivity, selectivity, and throughput categorized into “high”, “moderate”, and “low”. This allows for the comparison of methods used for each quantitation study presented in Table 1. Four studies (IX, XI, XIII-A and XVII) showed high sensitivity (limit of detection (LOD) ≤ 0.01–0.1 µg/mL) for all quantified compounds, while no LOD was determined in six studies. Most studies were assessed with moderate selectivity, as quantitation was performed using an UV detector. Four studies (VII, XIV, XIII-A and XVII) were attributed to high throughput since the method run time was ≤15 min per sample. Only study XVII was categorized as ‘high’ in all three categories.

Among lipophilic compounds, macamides and macaenes are most characteristic and abundant, often regarded as chemotaxonomic markers of maca [23]. The most frequently quantified macamide is **1**, followed by **6** and **7**, typically ranging from 0.1 to 1.2 mg/g dry weight [19] depending on root color and extraction method. Macaenes occur in similar concentrations [27] and may contribute to the plant’s adaptogenic effects. Chen et al. established a method for the simultaneous quantitation of 13 different macamides [56].

With respect to glucosinolates, compounds **26** and **27** are most commonly quantified. This compound class—particularly aromatic glucosinolates like glucotropaeolin (**28**)—represents a major group of polar constituents, occurring at levels of 1 to 4 mg/g dry root [60]. Glucosinolates are enzymatically hydrolyzed into isothiocyanates, which have demonstrated bioactivity in cancer chemoprevention and hormonal modulation [62].

Only few studies have focused on the quantitation of alkaloids, likely due to their occurrence as minor constituents, requiring sensitive detectors such as MS. For example, lepidiline A (**90**) and lepidiline B (**91**), though typically present <0.05 mg/g, are of pharmacological interest because of their potential neuroprotective and antioxidant effects [48].

In addition, sterols such as β-sitosterol, campesterol, and stigmasterol are found in minor amounts (0.01–0.1 mg/g), along with lignans and flavonoids [1]. To date, no studies have reported the quantitation of maca’s thiohydantoins.

The complex secondary metabolite profile of *L. meyenii* highly depends on ecotype and cultivation area (see chapter 4) [11]. In addition, post-harvest processing has a significant impact, with air dried hypocotyls showing higher macamide quantities than freeze dried hypocotyls, demonstrating the importance of the right drying process for the formation of macamides [55,63].

Currently, there are no official pharmacopeial monographs available for *L. meyenii*. However, due to its growing global popularity and use in nutraceuticals, several regulatory bodies have initiated efforts to establish quality standards. The United States Pharmacopeia (USP) is actively developing a dedicated monograph for maca root powder (https://doi.usp.org/USPNF/USPNF_M13115_10101_01.html, accessed 3 December 2025). The current draft includes identification procedures based on HPLC and proposes analytical standards for key marker compounds. These include the macamides *N*-benzylhexadecanamide (**1**), *N*-benzyl-(9Z,12Z)-octadecadienamide (**6**) and (9*Z*,12*Z*,15*Z*)-*N*-(phenylmethyl)-9,12,15-octadecatrienamide (**7**), as well as the glucosinolates glucotropaeolin (**28**), glucolimnanthin (**29**), sinalbin (**30**) and glucolepigramin (**31**).

### 3.3. Chemometric Methods Applied for Qualitative and Quantitative Analyses

Chemometric methods refer to the application of statistical and multivariate mathematical techniques to extract meaningful information from complex chemical data. In the analysis of *L. meyenii*, chemometrics has become an essential tool for interpreting the large and multidimensional datasets generated by advanced analytical instruments such as HPLC-DAD, LC-MS/MS, GC-MS, and NMR [16,23,64]. These methods are particularly valuable when dealing with natural products like maca, whose chemical composition might be influenced by numerous variables, including ecotype, altitude, harvest season, and post-harvest processing.

In qualitative analyses, chemometric techniques such as principal component analysis (PCA) and hierarchical cluster analysis (HCA) are widely employed for sample classification, authentication, and detection of adulterations. For example, PCA applied to *L. meyenii* metabolomic datasets has shown that the growing site exerts a stronger influence on the chemical profile than either ecotype or cultivation history. In one study, 72 maca samples from multiple Andean locations were analyzed using LC-MS-based metabolomics to generate the dataset supporting this conclusion [17]. Another investigation analyzed 60 samples representing different ecotypes and processing methods, confirming that geographic origin is the primary source of variability in the metabolome [65]. Similarly, a study of 48 samples across three ecotypes and several cultivation sites further demonstrated that site-specific environmental factors outweigh genetic differences in determining metabolite composition [16]. These studies highlight the importance of adequately sized, well-characterized datasets in chemometric analyses to reliably assess factors affecting maca’s chemical composition.

In this context, chemometrics enhances the discriminatory power of chromatographic or spectroscopic data by reducing dimensionality and highlighting patterns not readily apparent through visual inspection alone.

In quantitative studies, partial least squares regression (PLS) and variations of this statistical technique are commonly used to correlate chemical profiles with bioactivity data or quality parameters. For instance, PLS-DA can model the relationship between macamide concentrations and geographical origins [66], enabling predictive assessments of efficacy. The results thus provide potential evidence for the relationships between environmental or other factors and distribution of macamides.

Furthermore, supervised classification techniques such as orthogonal partial least squares-discriminant analysis (OPLS-DA) provide robust models for distinguishing between authentic and fake products or between raw and processed maca materials [15].

Chemometric approaches are increasingly recognized as valuable tools for quality control of complex herbal and natural products, supporting industrial applications such as method optimization, batch-to-batch consistency evaluation, and process control [65]. For example, multivariate statistical analyses, including principal component analysis and multivariate control charts, have been successfully applied to chromatographic fingerprints of botanical drug products to monitor hundreds of production batches, track variability, and ensure reproducible product quality during manufacturing processes [67]. Beyond batch control, chemometrics has been widely used to authenticate herbal medicines and discriminate between species, geographic origins, and processing methods based on phytochemical profiles, thereby enhancing reliability in industrial quality assurance workflows [68].

From a regulatory perspective, integrating chemometric evaluation with chromatographic and spectroscopic data is increasingly emphasized in quality control guidelines for herbal supplements and natural products. Such approaches enable the deconvolution of complex datasets, facilitate comprehensive characterization, and support compliance with stringent regulatory standards for safety, consistency, and product authentication [69]. Collectively, these examples illustrate the industrial relevance of chemometrics in standardizing maca-based nutraceuticals and other botanical products, bridging the gap between analytical research and practical quality assurance in commercial production.

When integrated with metabolomics workflows or fingerprinting strategies, chemometric analysis enhances the overall reliability and interpretability of phytochemical investigations, thereby contributing to better standardization, regulatory compliance, and consumer safety.

## 4. Maca Ecotypes and Geographical Differences

Maca hypocotyls occur in different color variations (yellow, red, black), and are predominately cultivated in Peru or China, motivating studies on how color and origin affect their phytochemical profiles. Early research findings suggested color-dependent differences in the phytochemical composition based on the quantitation of two macamides and one macaene [13]. However, later studies did not confirm this. In 2017, Chen et al. performed a principal component analysis (PCA) based on twelve macamides and macaenes from samples differing in color and origin [56]. Here, samples were distinguishable by origin but not by color. Similarly, PCA of ^1^H NMR spectra from various maca colors (yellow, violet, pink, lead) showed no clear color-based separation. A later PCA using flow injection mass spectrometry differentiated five color-origin groups, yet no specific ions could be linked to color or origin [16].

More recent quantification studies, however, suggest color does influence chemical composition. Meissner et al. [70] found significant differences in glucosinolate levels, though compound **27** occurred only in Peruvian samples, indicating origin may be more influential.

Peng et al. [57] reported notable variations in total macamide content based on both color and origin, with Chinese purple maca highest (1845.75 µg/g) and Peruvian black maca lowest (269.10 µg/g). These variations indicate that specific quantitation limits for each color-origin combination may be necessary for quality control. Further systematic studies are needed to clarify the roles of color and origin in determining macamide and glucosinolate profiles.

## 5. Potential Toxicity, Limitations, and Problems of Adulteration

Maca is generally considered safe for humans when consumed in moderate amounts (1.5–3 g/day), such as those typically found in food or dietary supplements [71]. Nevertheless, recommended daily intakes vary substantially among commercial products, and evidence-based, standardized dosing guidelines are lacking. Thus, safety issues remain relevant, particularly in the context of long-term use, high-dose supplementation, and product quality. Toxicity assessment of maca preparations commonly focuses on analytical markers such as glucosinolates and their degradation products (e.g., isothiocyanates), which may affect thyroid function at excessive intake levels and contribute to goitrogenic concerns [72,73]; alkaloids, including imidazole-, amidine-, and β-carboline-type compounds, due to their biological and potential neuroactive properties [11,74]; and heavy metals (e.g., lead, cadmium, arsenic) reflecting soil-related accumulation [75].

In *Caenorhabditis elegans*, high concentrations of aqueous maca extract reduced egg production, impaired vitellogenin expression, increased germline apoptosis, and altered lipid metabolism, indicating potential reproductive toxicity at elevated doses [76]. Animal studies in rats showed that administration of pre-gelatinized maca powder at doses up to 7.5 g/kg body weight for 28–90 days caused no toxicity or organ damage, indicating low acute and subchronic toxicity at levels far exceeding typical human consumption [77].

Regarding humans, toxicity symptoms reported in the literature are generally mild and transient at typical supplemental doses (2–3 g/day), with occasional gastrointestinal discomfort, headache, and irritability observed during 12-week oral administration; rare individual cases have described more serious events such as vaginal bleeding and a manic episode in single subjects, and mild gastrointestinal disturbances at higher intakes (e.g., ~5 g/day). At much larger or prolonged exposures, moderate alterations in blood pressure have been reported, but no consistent major organ toxicity or fatal outcomes have been documented in humans [11].

Most available safety and efficacy data are derived from short-term studies or traditional dietary use, while robust data on prolonged consumption or high-dose regimens remain limited [11]. A randomized, placebo-controlled human study administering 3 g/day either placebo, black, or red maca extract for 12 weeks showed acceptable tolerability and no serious adverse effects, although long-term safety was not evaluated [78].

Potential herb-drug interactions have not been systematically evaluated. Given reported endocrine-related, neuroactive, and metabolic effects of maca constituents, caution may be warranted when maca is consumed concomitantly with hormone-modulating therapies or centrally acting medications [11]. In addition, substantial chemical variability arising from differences in cultivars, geographic origin, cultivation conditions, and processing further complicates extrapolation of safety and efficacy data across products.

From a clinical perspective, many human studies investigating maca are characterized by small sample sizes, short intervention periods, heterogeneous study designs, and insufficient phytochemical characterization of test materials, which limits the strength, reproducibility, and generalizability of clinical conclusions [79].

These toxicological aspects, along with the pharmacological properties of maca, were recently examined in detail by Ulloa del Carpio and colleagues [11] and are therefore not discussed further here.

Beyond intrinsic safety and clinical limitations, economically motivated adulteration of maca products has become an increasing concern due to rising global demand and market value. Documented cases summarized by the American Botanical Council [https://umb.herbalgram.org/media/vvklxw20/bapp-babs-maca-cc-v6.pdf, accessed on 3 December 2025] include substitution with other powdered root crops, spiking with synthetic phosphodiesterase type 5 (PDE5) inhibitors [80], spiking with synthetically produced macamides, and mislabeling of maca cultivated outside Peru as genuine Peruvian-grown material.

In response, the U.S. Food and Drug Administration (FDA) has issued warning letters to manufacturers making unsubstantiated therapeutic claims about maca, although no specific enforcement actions targeting adulteration have been documented to date.

To address these issues, rigorous quality control and authentication strategies are essential. Analytical approaches such as chromatographic fingerprinting, LC–MS-based metabolite profiling, DNA barcoding using ITS sequences, and complementary spectroscopic methods have proven effective for detecting adulteration and ensuring product authenticity [14,80,81]. However, DNA barcoding may be limited in highly processed products or extracts due to DNA degradation or low DNA yields, potentially compromising reliable identification [82].

Overall, these considerations highlight the need for standardized analytical characterization, improved regulatory oversight, and well-designed long-term clinical studies using chemically well-defined maca preparations to ensure safety, appropriate dosing, reliable clinical interpretation, and consumer protection.

## 6. Methods of Literature Search

A comprehensive literature search was conducted to identify relevant analytical studies on *L. meyenii*. Initial searches were performed using the terms “maca” and “*Lepidium meyenii*” in PubMed, including references from the MEDLINE database. Additional searches were conducted through SciFinder, containing references from the MEDLINE, CAplus and ChemZent databases. In total, more than 150 publications dealing with the isolation of metabolites, qualitative and quantitative analyses, pharmacological and toxicological data found in PubMed and SciFinder since the beginning of records were reviewed. Therefore, keywords including “isolation”, “quantitative analysis”, “HPLC”, “LC-MS”, “bioactivity” and “toxicity” were combined with the terms “*Lepidium meyenii*” or “maca”. Google was used to locate commercial websites of maca sellers, and manual searches were carried out to retrieve relevant references from bibliographies of key articles. Selected articles were organized into categorized folders within the reference management program EndNote X9. The literature review includes sources published between 2000 up to December 2025.

## 7. Conclusions and Future Perspectives

*L. meyenii* (maca) continues to attract global interest due to its rich ethnopharmacological history, diverse phytochemical composition, and broad spectrum of potential health benefits. Advances in analytical techniques have significantly enhanced our understanding of maca’s secondary metabolites. In particular, macamides, macaenes, and glucosinolates, which serve as key markers for quality control, authentication, and potential pharmacological efficacy, are in the focus of quantitative studies. Despite the chemical variability among ecotypes, geographic origin appears to exert a greater influence on metabolite distribution, underscoring the need for well-designed cultivation and standardization practices. While HPLC-UV remains a widely employed technique for quantitation, the integration of UHPLC-MS and qNMR, combined with chemometric tools such as PCA and PLS, has enabled more robust profiling, discrimination, and prediction of biological activity. Interestingly, advanced techniques such as supercritical fluid extraction (SFE) and chromatography (SFC) remain underutilized in maca research. This might be primarily due to the high cost of instrumentation, the need for specialized technical expertise, and limited standardization of protocols for complex plant matrices despite the technology’s advantages in efficiency and selectivity in particular for lipophilic compounds from natural sources [83].

However, challenges persist due to the lack of official pharmacopeial monographs, increasing adulteration risks, and limited long-term safety data. Despite its uncertain toxicological properties, maca has been traditionally used as a herbal remedy without any related complications reported. Due to the increasing demand of maca, test guidelines need to be established to ensure the quality and safety of marketed products. Based on the pharmacological relevance reported by in vitro and in vivo studies, the quantitation of macamides is fundamental for quality control of maca containing products, which also allows us to draw conclusions about the ecotype, origin and proper drying procedures of the hypocotyls. HPLC-UV analysis appears to be the most common method, with several studies showing its applicability. This method can also be applied for the analysis of maca’s glucosinolates, which may serve as important markers to differentiate hypocotyls according to their origin. To achieve shorter run times, UHPLC represents a suitable alternative. Moreover, (U)HPLC-MS is required for the analysis of maca’s alkaloids as they are only present as minor constituents. To check for adulterations with synthetic drugs (i.e., PDE-5 inhibitors), simple and cost-effective methods should be applied for qualitative analysis. Therefore, the development of a TLC method, IR analysis or a simple chemical reaction with a color reagent might be a suitable approach. Future research might be directed towards the establishment of reference standards as validated marker compounds, more profound toxicological assessments, and the development of quality guidelines to support the safe and effective use of maca in phytomedicine and evidence-based nutraceuticals.

## Figures and Tables

**Table 2:** Comparative overview of sensitivity, selectivity, and throughput of quantitative analyses described in Table 1.

Study No.	Method Validation	Sensitivity ^a^	Selectivity ^b^	Throughput ^c^	Ref.
I-A	no	n. d. ^d^	moderate	low	[26]
I-B	no	n. d. ^d^	moderate-high	low	
II	partially	moderate-low	moderate	low	[27]
III	yes	moderate	moderate	moderate	[19]
IV	no	n. d. ^d^	moderate	low	[58]
V	no	n. d. ^d^	moderate	low	[13]
VI	yes	moderate-low	moderate	moderate	[56]
VII	yes	high-moderate	moderate-high	high	[54]
VIII	no	n. d. ^d^	high	low	[59]
IX	yes	high	moderate	low	[23]
X	yes	low	low	n. a. ^e^	[57]
XI	partially	high	moderate	low	[55]
XII	yes	low	moderate	moderate	[32]
XIII-A	yes	high	moderate	high	[30]
XIII-B	yes	moderate	high	moderate	
XIV	yes	high-moderate	high	high	[29]
XV	partially	n. d. ^d^	high	low	[60]
XVI	yes	moderate	moderate	moderate	[61]
XVII	yes	high	high	high	[3]

^a^ Sensitivity is ranked qualitatively based on reported LOD ranges: high (LOD ≤ 0.01–0.1 µg/mL); moderate (LOD 0.1–1 µg/mL); low (LOD ≥ 1 µg/mL). ^b^ Selectivity was categorized based on detection mode: high (MS/MS; high-resolution MS); moderate-high (single quadrupole MS); moderate (UV/DAD); low (NMR). ^c^ Throughput reflects chromatographic run time per sample: high (≤15 min/sample); moderate (15–30 min/sample); low (≥30 min/sample). ^d^ n. d., not determined. ^e^ n. a., not applicable.

## Data Availability

No new data was created in this study. Data sharing is not applicable to this article.

## Supplementary Materials

The following supporting information can be downloaded at: https://www.mdpi.com/article/10.3390/foods15020306/s1, Table S1: Reported secondary metabolites from maca (*L. meyenii*); Figure S1: Chemical structures of macamides known for maca (*L. meyenii*); Figure S2: Chemical structures of macaenes and fatty acids known for maca (*L. meyenii*); Figure S3: Chemical structures of glucosinolates known for maca (*L. meyenii*); Figure S4: Chemical structures of thiohydantoins and related metabolites known for maca (*L. meyenii*); Figure S5: Chemical structures of alkaloids known for maca (*L. meyenii*).

## Abbreviations

The following abbreviations are used in this manuscript:

DAD	Diode array detection
ESI	Electrospray ionization
GC-MS	Gas chromatography-mass spectrometry
HCA	Hierarchical cluster analysis
HPLC	High-performance liquid chromatography
IR	Infrared spectroscopy
LC-MS/MS	Liquid chromatography-tandem mass spectrometry
MS	Mass spectrometry
NMR	Nuclear magnetic resonance
OPLS-DA	Orthogonal partial least squares-discriminant analysis
PCA	Principal component analysis
PLS	Partial least squares regression
TLC	Thin layer chromatography
U(H)PLC	Ultra-(high)performance liquid chromatography
