# Supplementary material for: Maca (Lepidium meyenii) as a Functional Food and Dietary Supplement: A Review on Analytical Studies
Source: Foods. 2026 Jan 14;15(2):306. doi: 10.3390/foods15020306 (PMC12840177; doi:10.3390/foods15020306)
Supplement: Supplementary file 1 [file foods-15-00306-s001.zip › foods-4056604-supplementary.pdf]

foods

MDPI

Supplementary Material

Maca (*Lepidium meyenii*) as a Functional Food and Dietary Supplement: A Review on Analytical Studies

Andreas Wasilewicz * and Ulrike Grienke

Division of Pharmacognosy, Department of Pharmaceutical Sciences, Faculty of Life Sciences, University of Vienna, Josef-Holaubek-Platz 2, 1090 Vienna, Austria;
* Correspondence: andreas.wasilewicz@univie.ac.at

Contents

Table S1 Reported secondary metabolites from maca (*L. meyenii*)..........2

Figure S1 Chemical structures of macamides known for maca (*L. meyenii*)..........9

Figure S2 Chemical structures of macaenes and fatty acids known for maca (*L. meyenii*)..........10

Figure S3 Chemical structures of glucosinolates known for maca (*L. meyenii*)..........10

Figure S4 Chemical structures of thiohydantoins and related metabolites known for maca (*L. meyenii*)..........11

Figure S5 Chemical structures of alkaloids known for maca (*L. meyenii*)..........12

References..........13

Table S1 Reported secondary metabolites from maca (*L. meyenii*).

Cpd no.	Name	CAS number	Compound class	Molecular mass	Molecular formula	Ref.
1	*N*-benzylhexadecanamide	74058-71-2	macamide	345.56	$C_{23}H_{39}NO$	[1]
2	*N*-(Phenylmethyl)pentadecanamide	1572037-13-8	macamide	331.54	$C_{22}H_{37}NO$	[2]
3	*N*-(Phenylmethyl)heptadecanamide	883715-19-3	macamide	359.60	$C_{24}H_{41}NO$	[2]
4	*N*-(Phenylmethyl)octadecanamide	5327-45-7	macamide	373.63	$C_{25}H_{43}NO$	[2]
5	(9*Z*)-*N*-(Phenylmethyl)-9-octadecenamide	101762-87-2	macamide	371.60	$C_{25}H_{41}NO$	[3]
6	(9*Z*,12*Z*)-*N*-(Phenylmethyl)-9,12-octadecadienamide	18286-71-0	macamide	369.58	$C_{25}H_{39}NO$	[3]
7	(9*Z*,12*Z*,15*Z*)-*N*-(Phenylmethyl)-9,12,15-octadecatrienamide	883715-18-2	macamide	367.57	$C_{25}H_{37}NO$	[4]
8	(6*E*,8*E*)-5-Oxo-*N*-(phenylmethyl)-6,8-octadecadienamide	405906-95-8	macamide	383.57	$C_{25}H_{37}NO_2$	[1]
9	(12*Z*)-9-Oxo-*N*-(phenylmethyl)-12-octadecenamide	847361-88-0	macamide	385.58	$C_{25}H_{39}NO_2$	[5]
10	(12*Z*,15*Z*)-9-Oxo-*N*-(phenylmethyl)-12,15-octadecadienamide	847361-90-4	macamide	383.57	$C_{25}H_{37}NO_2$	[5]
11	(10*E*,12*E*)-9-Oxo-*N*-(phenylmethyl)-10,12-octadecadienamide	2155902-81-9	macamide	383.57	$C_{25}H_{37}NO_2$	[6]
12	(10*E*,12*Z*)-9-Oxo-*N*-(phenylmethyl)-10,12-octadecadienamide	2155902-82-0	macamide	383.57	$C_{25}H_{37}NO_2$	[6]
13	(9*E*,11*E*)-13-Oxo-*N*-(phenylmethyl)-9,11-octadecadienamide	847361-92-6	macamide	383.57	$C_{25}H_{37}NO_2$	[5]

Cpd no.	Name	CAS number	Compound class	Molecular mass	Molecular formula	Ref.
14	*N*-[(3-Methoxyphenyl)methyl]hexadecanamide	847361-96-0	macamide	357.59	$C_{24}H_{41}NO_2$	[5]
15	*N*-[(3-Methoxyphenyl)methyl]octadecanamide	1429659-99-3	macamide	403.65	$C_{26}H_{45}NO_2$	[2]
16	(9*Z*,12*Z*)-*N*-[(3-Methoxyphenyl)methyl]-9,12-octadeca-dienamide	883715-22-8	macamide	399.62	$C_{26}H_{41}NO_2$	[2]
17	(9*Z*,12*Z*,15*Z*)-*N*-[(3-Methoxyphenyl)methyl]-9,12,15-octadecatrienamide	883715-23-9	macamide	397.60	$C_{26}H_{39}NO_2$	[2]
18	*N*-[(3,4-Dimethoxyphenyl)methyl]hexadecanamide	1638526-09-6	macamide	405.61	$C_{25}H_{43}NO_3$	[7]
19	(9*Z*)-*N*-[(3,4-Dimethoxyphenyl)methyl]-9-octadecena-mide	2260819-10-9	macamide	431.65	$C_{27}H_{43}NO_3$	[8]
20	*N*-(Phenylmethyl)tetracosanamide	1638527-02-2	macamide	457.78	$C_{31}H_{55}NO$	[7]
21	(15*Z*)-*N*-(Phenylmethyl)-15-tetracosenamide	847361-94-8	macamide	455.76	$C_{31}H_{53}NO$	[5]
22	(6*E*,8*E*)-5-Oxo-6,8-octadecadienoic acid	405906-96-9	macaene	294.43	$C_{18}H_{30}O_3$	[1]
23	(10*E*,12*Z*)-9-Oxo-10,12-octadecadienoic acid	54232-59-6	macaene	294.43	$C_{18}H_{30}O_3$	[6]
24	(10*E*,12*E*)-9-Oxo-10,12-octadecadienoic acid	54232-58-5	macaene	294.43	$C_{18}H_{30}O_3$	[6]
25	(9*Z*,11*E*)-13-Oxo-9,11-octadecadienoic acid	54739-30-9	macaene	294.43	$C_{18}H_{30}O_3$	[9]
26	Linoleic acid	60-33-3	fatty acid	280.45	$C_{18}H_{32}O_2$	[10]
27	Linolenic acid	463-40-1	fatty acid	278.43	$C_{18}H_{30}O_2$	[10]
28	Glucotropaeolin	499-26-3	aromatic glucosinolate	409.42	$C_{14}H_{19}NO_9S_2$	[11]

https://doi.org/10.3390/xxxxx

Cpd no.	Name	CAS number	Compound class	Molecular mass	Molecular formula	Ref.
29	Glucolimnathin	111810-95-8	aromatic glucosinolate	439.45	$C_{15}H_{21}NO_{10}S_2$	[11]
30	Glucosinalibin	19253-84-0	aromatic glucosinolate	425.42	$C_{14}H_{19}NO_{10}S_2$	[12]
31	Glucoepigramin	59204-64-7	aromatic glucosinolate	425.42	$C_{14}H_{19}NO_{10}S_2$	[13]
32	Glucoaubrietin	499-27-4	aromatic glucosinolate	439.45	$C_{15}H_{21}NO_{10}S_2$	[14]
33	Glucoallysin	499-37-6	aliphatic glucosinolate	451.52	$C_{13}H_{25}NO_{10}S_3$	[12]
34	Glucoraphanin	21414-41-5	aliphatic glucosinolate	437.49	$C_{12}H_{23}NO_{10}S_3$	[15]
35	Glucobrassicanapin	19041-10-2	aliphatic glucosinolate	387.42	$C_{12}H_{21}NO_9S_2$	[12]
36	Glucobrassicin	4356-52-9	indole glucosinolate	448.46	$C_{16}H_{20}N_2O_9S_2$	[12]
37	4-Hydroxyglucobrassicin	83327-20-2	indole glucosinolate	464.46	$C_{16}H_{20}N_2O_{10}S_2$	[12]
38	4-Methoxyglucobrassicin	83327-21-3	indole glucosinolate	478.49	$C_{17}H_{22}N_2O_{10}S_2$	[12]
39	Macathiohydantoin A	934748-86-4	thiohydantoin	246.33	$C_{13}H_{14}N_2OS$	[16]
40	Macathiohydantoin B	2114451-93-1	thiohydantoin	262.33	$C_{13}H_{14}N_2O_2S$	[16]
41	Macathiohydantoin C	2113669-98-8	thiohydantoin	276.35	$C_{14}H_{16}N_2O_2S$	[16]
42	Macathiohydantoin D	2113669-99-9	thiohydantoin	262.33	$C_{13}H_{14}N_2O_2S$	[16]
43	Macathiohydantoin E	2114451-94-2	thiohydantoin	278.33	$C_{13}H_{14}N_2O_3S$	[16]

https://doi.org/10.3390/xxxxx

Cpd no.	Name	CAS number	Compound class	Molecular mass	Molecular formula	Ref.
44	Macathiohydantoin F	2114451-95-3	thiohydantoin	276.35	$C_{14}H_{16}N_2O_2S$	[16]
45	Macathiohydantoin G	2114451-96-4	thiohydantoin	292.35	$C_{14}H_{16}N_2O_3S$	[16]
46	Macathiohydantoin H	2114451-97-5	thiohydantoin	340.44	$C_{19}H_{20}N_2O_2S$	[16]
47	Macathiohydantoin I	2114451-98-6	thiohydantoin	340.44	$C_{19}H_{20}N_2O_2S$	[16]
48	Macathiohydantoin J	2114451-99-7	thiohydantoin	326.41	$C_{18}H_{18}N_2O_2S$	[16]
49	Macathiohydantoin K	2114452-00-3	thiohydantoin	356.44	$C_{19}H_{20}N_2O_3S$	[16]
50	Macathiohydantoin L	n. a.	thiohydantoin	276.35	$C_{14}H_{16}N_2O_2S$	[17]
51	Macathiohydantoin M	n. a.	thiohydantoin	338.50	$C_{15}H_{18}N_2OS_3$	[17]
52	Macathiohydantoin N	n. a.	thiohydantoin	368.53	$C_{16}H_{20}N_2O_2S_3$	[17]
53	(+)-Macathiohydantoin O	3035416-79-3	thiohydantoin	306.38	$C_{15}H_{18}N_2O_3S$	[17]
54	Macathiohydantoin P	n. a.	thiohydantoin	310.42	$C_{18}H_{18}N_2OS$	[18]
55	Macathiohydantoin Q	n. a.	thiohydantoin	326.41	$C_{18}H_{18}N_2O_2S$	[18]
56	Macathiohydantoin R	n. a.	thiohydantoin	356.44	$C_{19}H_{20}N_2O_3S$	[18]
57	Macahydantoin A	2120391-96-8	thiohydantoin	260.36	$C_{14}H_{16}N_2OS$	[16]
58	Macahydantoin B	2129094-21-7	thiohydantoin	306.38	$C_{15}H_{18}N_2O_3S$	[19]

Cpd no.	Name	CAS number	Compound class	Molecular mass	Molecular formula	Ref.
59	(+)-Meyeniin A	2091284-00-1	thiohydantoin	322.40	$C_{14}H_{14}N_2O_3S_2$	[20]
60	(+)-Meyeniin B	2091283-99-5	thiohydantoin	278.39	$C_{13}H_{14}N_2OS_2$	[20]
61	(+)-Meyeniin C	2095122-95-3	thiohydantoin	308.41	$C_{14}H_{16}N_2O_2S_2$	[20]
62	(+)-Meyeniin D	3035416-80-6	thiohydantoin	310.39	$C_{13}H_{14}N_2O_3S_2$	[17]
63	(4R,6R/S) 6-Hydroxyl macathiohydantoin C	n. a.	thiohydantoin	292.35	$C_{14}H_{16}N_2O_3S$	[21]
64	(4*S*,6*S*)-6-Hydroxyl macathiohydantoin A	n. a.	thiohydantoin	262.33	$C_{13}H_{14}N_2O_2S$	[21]
65	(±)-Lepithiohydantoin A	n. a.	thiohydantoin	554.76	$C_{26}H_{26}N_4O_2S_4$	[22]
66	(±)-Lepithiohydantoin B	n. a.	thiohydantoin	570.76	$C_{26}H_{26}N_4O_3S_4$	[22]
67	(±)-Lepithiohydantoin C	n. a.	thiohydantoin	584.79	$C_{27}H_{28}N_4O_3S_4$	[22]
68	(±)-Lepithiohydantoin D	n. a.	thiohydantoin	538.70	$C_{26}H_{26}N_4O_3S_3$	[22]
69	Macahydantoin C	n. a.	hydantoin	246.27	$C_{13}H_{12}N_2O_3$	[23]
70	Macahydantoin D	2172357-46-7	hydantoin	230.27	$C_{13}H_{14}N_2O_2$	[23]
71	Macaurea A	n. a.	urea derivative	337.42	$C_{20}H_{23}N_3O_2$	[23]
72	Macathioamide A	n. a.	thioamide	284.38	$C_{16}H_{16}N_2OS$	[23]
73	Macapyrrolin A	2417119-52-7	pyrrol alkaloid	229.27	$C_{14}H_{15}NO_2$	[24]

Cpd no.	Name	CAS number	Compound class	Molecular mass	Molecular formula	Ref.
74	Macapyrrolin B	2417119-53-8	pyrrol alkaloid	245.27	$C_{14}H_{15}NO_3$	[24]
75	Macapyrrolin C	1802248-79-8	pyrrol alkaloid	215.25	$C_{13}H_{13}NO_2$	[25]
76	Macapyrrolin D[a]	2839456-58-3	pyrrol alkaloid	259.30	$C_{15}H_{17}NO_3$	[26]
77	Macapyrrolin E[b]	2839456-59-4	pyrrol alkaloid	245.27	$C_{14}H_{15}NO_3$	[26]
78	Macapyrrolin E[b]	n. a.	pyrrol alkaloid	257.29	$C_{15}H_{15}NO_3$	[27]
79	Macapyrrolin F	n. a.	pyrrol alkaloid	287.32	$C_{16}H_{17}NO_4$	[27]
80	Macapyrrolin G	n. a.	pyrrol alkaloid	243.31	$C_{15}H_{17}NO_2$	[27]
81	Macapyrrolin D[a]	n. a.	pyrrol alkaloid	333.39	$C_{21}H_{19}NO_3$	[27]
82	Macapyrrolin J	n. a.	pyrrol alkaloid	215.25	$C_{13}H_{13}NO_2$	[27]
83	n. a.	1178318-81-4	pyrrol alkaloid	215.25	$C_{13}H_{13}NO_2$	[27]
84	Lepipyrrolin A	n. a.	pyrrol alkaloid	412.49	$C_{26}H_{24}N_2O_3$	[27]
85	Lepipyrrolin B	n. a.	pyrrol alkaloid	442.52	$C_{27}H_{26}N_2O_4$	[27]
86	(5*S*)-Macapyrrolidone A	208655-69-0	pyrrolidine alkaloid	217.26	$C_{13}H_{15}NO_2$	[26]
87	(5*R*)-Macapyrrolidone A	2839456-52-7	pyrrolidine alkaloid	217.26	$C_{13}H_{15}NO_2$	[26]
88	(5*S*)-Macapyrrolidone B	2839456-56-1	pyrrolidine alkaloid	247.29	$C_{14}H_{17}NO_3$	[26]

Cpd no.	Name	CAS number	Compound class	Molecular mass	Molecular formula	Ref.
89	(5*R*)-Macapyrrolidone B	2839456-53-8	pyrrolidine alkaloid	247.29	$C_{14}H_{17}NO_3$	[26]
90	Lepidiline A	596093-98-0	imidazole alkaloid	277.39	$C_{19}H_{21}N_2^+$	[28]
91	Lepidiline B	596093-97-9	imidazole alkaloid	291.42	$C_{20}H_{23}N_2^+$	[28]
92	Lepidiline C	1236292-70-8	imidazole alkaloid	307.42	$C_{20}H_{23}N_2O^+$	[29]
93	Lepidiline D	1236292-71-9	imidazole alkaloid	321.44	$C_{21}H_{25}N_2O^+$	[29]
94	Lepidiline E	2801646-86-4	imidazole alkaloid	227.33	$C_{15}H_{19}N_2^+$	[25]
95	Lepidiline F	2801647-52-7	imidazole alkaloid	257.36	$C_{16}H_{21}N_2O^+$	[25]
96	Lepidiline G	2801648-67-7	imidazole alkaloid	215.32	$C_{14}H_{19}N_2^+$	[25]
97	(1*R*,3*S*)-1-Methyl-1,2,3,4-tetrahydro-β-carboline-3-carboxylic acid	42438-72-2	β-carboline alkaloid	230.26	$C_{13}H_{14}N_2O_2$	[30]
98	(1*S*,3*S*)-1-Methyl-1,2,3,4-tetrahydro-β-carboline-3-carboxylic acid	40678-46-4	β-carboline alkaloid	230.26	$C_{13}H_{14}N_2O_2$	[30]
99	1,2,3,4-Tetrahydro-*β*-carboline-3-carboxylic acid	6052-68-2	β-carboline alkaloid	216.24	$C_{12}H_{12}N_2O_2$	[25]
100	Macaridine	405914-36-5	pyridine alkaloid	215.25	$C_{13}H_{13}NO_2$	[1]
101	*N*,*N*′-Dibenzylformamidine	4636-51-5	amidine alkaloid	224.30	$C_{15}H_{16}N_2$	[25]
102	*N*,*N*′-Dibenzylacetamidine	41992-33-0	amidine alkaloid	238.33	$C_{16}H_{18}N_2$	[25]

n. a., not available; [a,b], compounds share the same name.

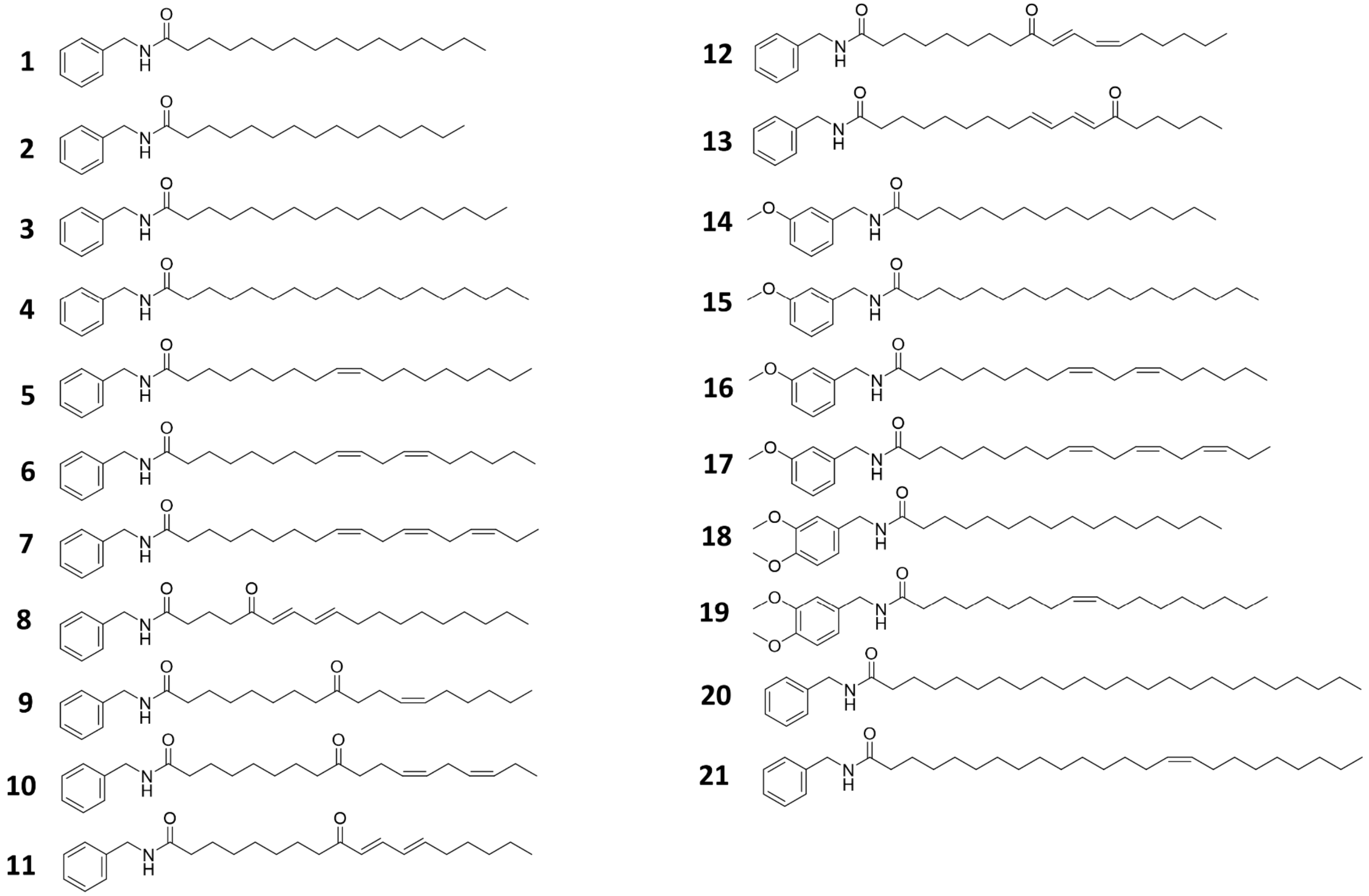

Figure S1. Chemical structures of macamides known for maca (*L. meyenii*)

https://doi.org/10.3390/xxxxx

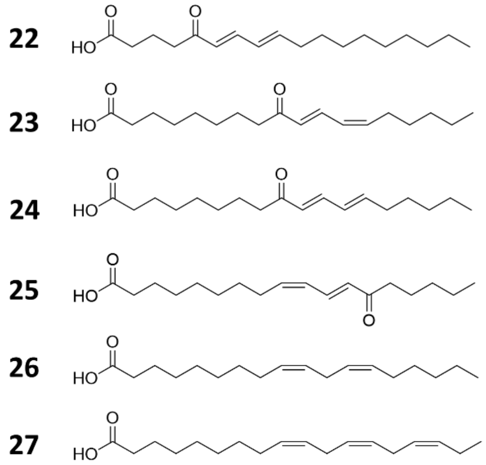

Figure S2. Chemical structures of macaenes and fatty acids known for maca (*L. meyenii*)

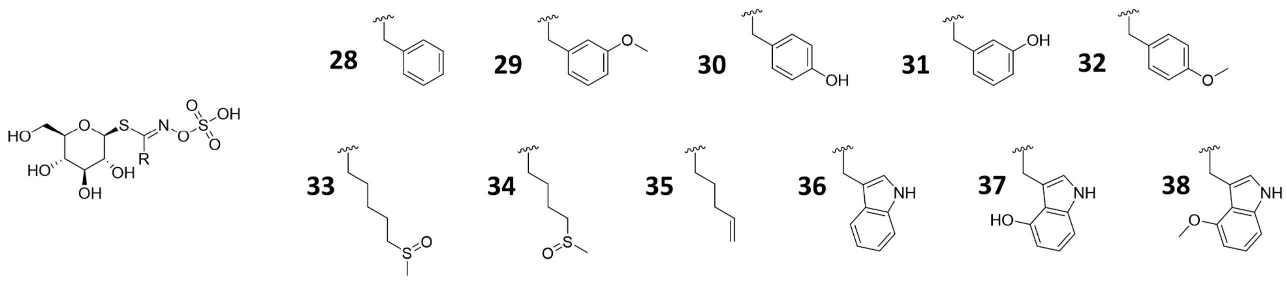

Figure S3. Chemical structures of glucosinolates known for maca (*L. meyenii*)

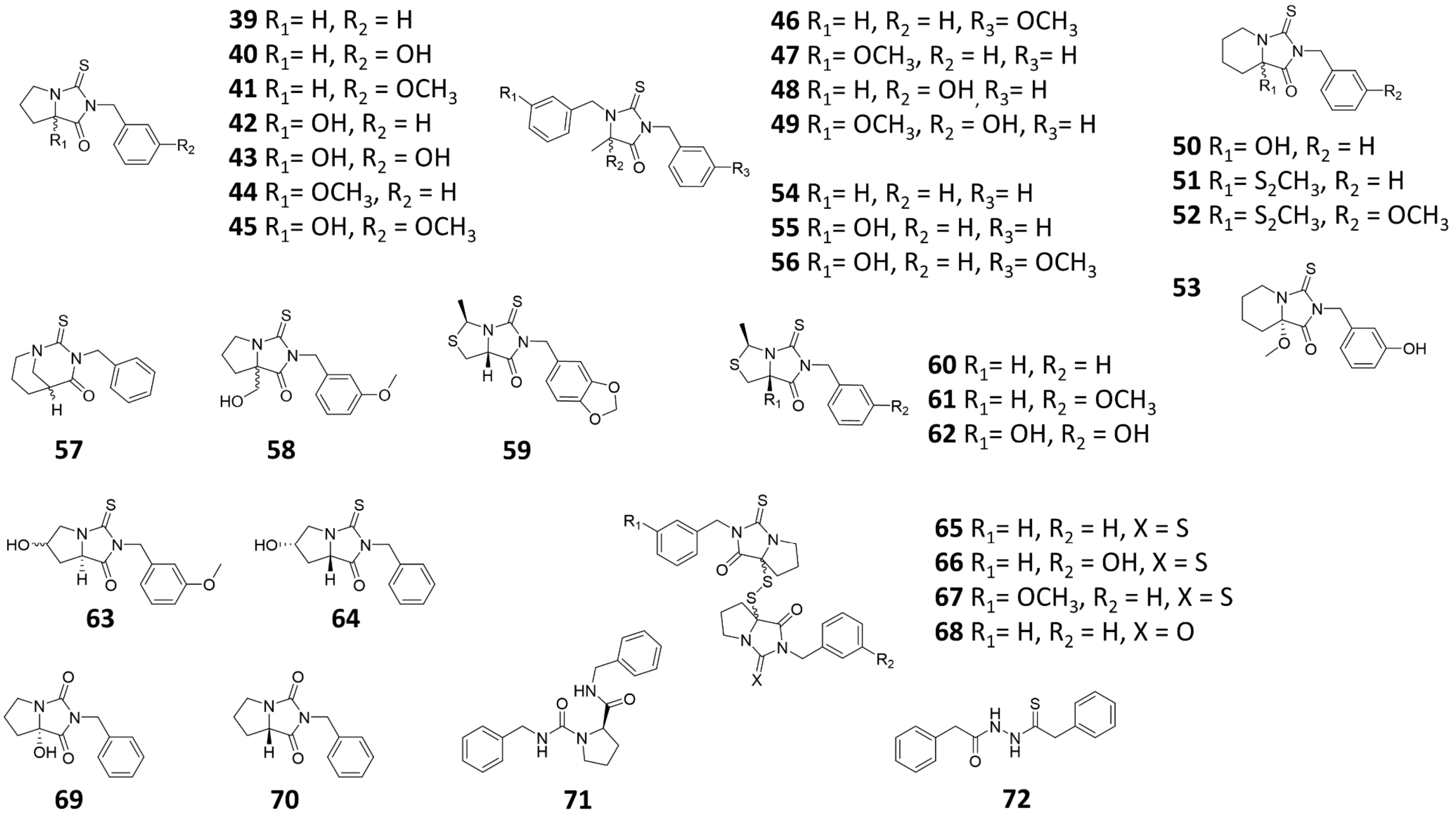

Figure S4. Chemical structures of thiohydantoins and related metabolites known for maca (*L. meyenii*)

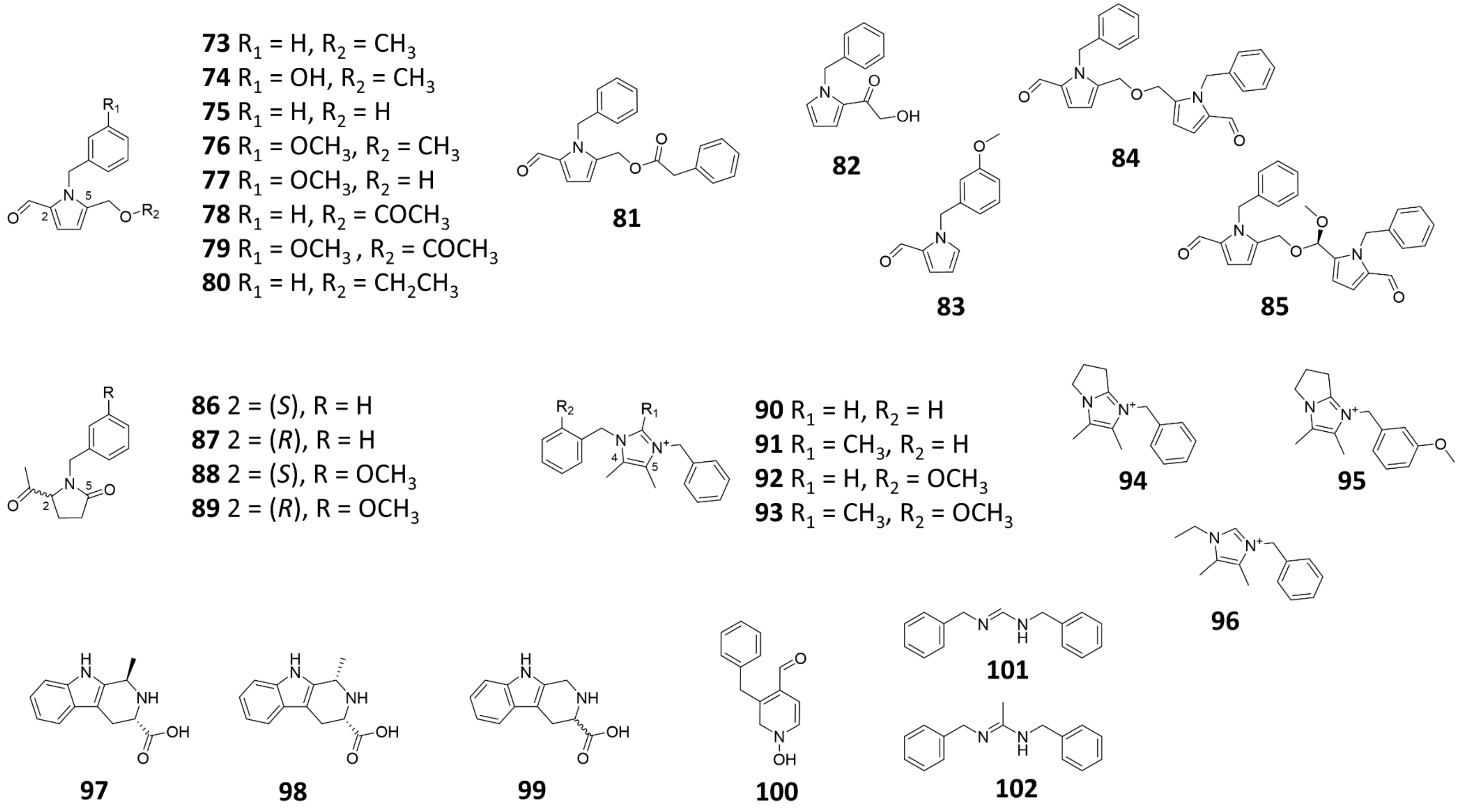

Figure S5. Chemical structures of alkaloids known for maca (*L. meyenii*)

References

1. Muhammad, I.; Zhao, J.; Dunbar, D.C.; Khan, I.A. Constituents of *Lepidium meyenii* 'maca'. *Phytochemistry* **2002**, *59*, 105-110. doi:10.1016/s0031-9422(01)00395-8
2. Chen, S.X.; Li, K.K.; Pubu, D.; Jiang, S.P.; Chen, B.; Chen, L.R.; Yang, Z.; Ma, C.; Gong, X.J. Optimization of ultrasound-assisted extraction, HPLC and UHPLC-ESI-Q-TOF-MS/MS analysis of main macamides and macaenes from maca (cultivars of *Lepidium meyenii* Walp). *Molecules* **2017**, *22*. doi:10.3390/molecules22122196
3. McCollom, M.M.; Villinski, J.R.; McPhail, K.L.; Craker, L.E.; Gafner, S. Analysis of macamides in samples of maca (*Lepidium meyenii*) by HPLC-UV-MS/MS. *Phytochem. Anal.* **2005**, *16*, 463-469. doi:10.1002/pca.871
4. Zhong, J.L.; Yan, H.; Xu, H.D.; Muhammad, N.; Yan, W.D. Preparation from *Lepidium meyenii* Walpers using high-speed countercurrent chromatography and thermal stability of macamides in air at various temperatures. *J. Pharm. Biomed. Anal.* **2019**, *164*, 768-776. doi:10.1016/j.jpba.2018.11.041
5. Zhao, J.; Muhammad, I.; Dunbar, D.C.; Mustafa, J.; Khan, I.A. New alkamides from maca (*Lepidium meyenii*). *J. Agric. Food Chem.* **2005**, *53*, 690-693. doi:10.1021/jf048529t
6. Xia, C.; Deng, J.; Chen, J.; Zhu, Y.; Song, Y.; Zhang, Y.; Li, H.; Lin, C. Simultaneous determination of macaenes and macamides in maca using an HPLC method and analysis using a chemometric method (HCA) to distinguish maca origin. *Rev. Bras. Farmacogn.* **2019**, *29*, 702-709. doi:10.1016/j.bjp.2019.05.009
7. Chain, F.E.; Grau, A.; Martins, J.C.; Catalán, C.A.N. Macamides from wild 'Maca', *Lepidium meyenii* Walpers (Brassicaceae). *Phytochem. Lett.* **2014**, *8*, 145-148. doi:10.1016/j.phytol.2014.03.005
8. Ye, Y.Q.; Ma, Z.H.; Yang, Q.F.; Sun, Y.Q.; Zhang, R.Q.; Wu, R.F.; Ren, X.; Mu, L.J.; Jiang, Z.Y.; Zhou, M. Isolation and synthesis of a new benzylated alkamide from the roots of *Lepidium meyenii*. *Nat. Prod. Res.* **2019**, *33*, 2731-2737. doi:10.1080/14786419.2018.1499633
9. Xia, C.; Deng, J.; Pan, Y.; Lin, C.; Zhu, Y.; Xiang, Z.; Li, W.; Chen, J.; Zhang, Y.; Zhu, B.; et al. Comprehensive profiling of macamides and fatty acid derivatives in maca with different postharvest drying processes using UPLC-QTOF-MS. *ACS Omega* **2021**, *6*, 24484-24492. doi:10.1021/acsomega.1c02926
10. Ganzera, M.; Zhao, J.; Muhammad, I.; Khan, I.A. Chemical profiling and standardization of *Lepidium meyenii* (Maca) by reversed phase high performance liquid chromatography. *Chem. Pharm. Bull.* **2002**, *50*, 988-991. doi:10.1248/cpb.50.988
11. Piacente, S.; Carbone, V.; Plaza, A.; Zampelli, A.; Pizza, C. Investigation of the tuber constituents of maca (*Lepidium meyenii* Walp.). *J. Agric. Food Chem.* **2002**, *50*, 5621-6525. doi:10.1021/jf020280x
12. Yábar, E.; Pedreschi, R.; Chirinos, R.; Campos, D. Glucosinolate content and myrosinase activity evolution in three maca (*Lepidium meyenii* Walp.) ecotypes during preharvest, harvest and postharvest drying. *Food Chem.* **2011**, *127*, 1576-1583. doi:10.1016/j.foodchem.2011.02.021
13. Xu, Q.; Monagas, M.J.; Kassymbek, Z.K.; Belsky, J.L. Controlling the quality of maca (*Lepidium meyenii*) dietary supplements: Development of compendial procedures for the determination of intact glucosinolates in maca root powder products. *J. Pharm. Biomed. Anal.* **2021**, *199*, 114063. doi:10.1016/j.jpba.2021.114063
14. Li, G.; Ammermann, U.; Quirós, C.F. Glucosinolate contents in maca (*Lepidium peruvianum* Chacón) seeds, sprouts, mature plants and several derived commercial products. *Econ. Bot.* **2001**, *55*, 255-262. doi:10.1007/BF02864563
15. Clement, C.; Diaz Grados, D.A.; Avula, B.; Khan, I.A.; Mayer, A.C.; Ponce Aguirre, D.D.; Manrique, I.; Kreuzer, M. Influence of colour type and previous cultivation on secondary metabolites in hypocotyls and leaves of maca (*Lepidium meyenii* Walpers). *J. Sci. Food Agric.* **2010**, *90*, 861-869. doi:10.1002/jsfa.3896
16. Yu, M.-Y.; Qin, X.-J.; Peng, X.-R.; Wang, X.; Tian, X.-X.; Li, Z.-R.; Qiu, M.-H. Macathiohydantoins B–K, novel thiohydantoin derivatives from *Lepidium meyenii*. *Tetrahedron* **2017**, *73*, 4392-4397. doi:10.1016/j.tet.2017.05.096
17. Zhang, R.; Liu, J.; Yan, H.; Peng, X.; Zhang, L.; Qiu, M. Macathiohydantoin L, a novel thiohydantoin bearing a thioxohexahydroimidazo [1,5-a] pyridine moiety from maca (*Lepidium meyenii* Walp.). *Molecules* **2021**, *26*. doi:10.3390/molecules26164934
18. Geng, H.-C.; Wang, X.-S.; Liao, Y.-J.; Qiu, S.-Y.; Fang, H.-X.; Chen, X.-L.; Wang, Y.-M.; Zhou, M. Macathiohydantoins P–R, three new thiohydantoin derivatives from maca (*Lepidium meyenii*). *Phytochem. Lett.* **2022**, *51*, 67-70. doi:10.1016/j.phytol.2022.07.007
19. Yu, M.-Y.; Qin, X.-J.; Shao, L.-D.; Peng, X.-R.; Li, L.; Yang, H.; Qiu, M.-H. Macahydantoins A and B, two new thiohydantoin derivatives from maca (*Lepidium meyenii*): Structural elucidation and concise synthesis of macahydantoin A. *Tetrahedron Lett.* **2017**, *58*, 1684-1686. doi:10.1016/j.tetlet.2017.03.038

20. Zhou, M.; Ma, H.Y.; Liu, Z.H.; Yang, G.Y.; Du, G.; Ye, Y.Q.; Li, G.P.; Hu, Q.F. (+)-Meyeniins A-C, novel hexahydroimidazo[1,5-c]thiazole derivatives from the tubers of *Lepidium meyenii*: Complete structural elucidation by biomimetic synthesis and racemic crystallization. *J. Agric. Food Chem.* **2017**, *65*, 1887-1892. doi:10.1021/acs.jafc.6b05805
21. Liu, W.-X.; Li, J.; Liao, C.-J.; Yang, F.-W.; Geng, H.-C.; Zhou, M. New thiohydantoin derivatives from the roots of *Lepidium meyenii*. *Phytochem. Lett.* **2025**, *67*, 102964. doi:10.1016/j.phytol.2025.102964
22. Peng, X.-R.; Zhang, R.-R.; Liu, J.-H.; Li, Z.-R.; Zhou, L.; Qiu, M.-H. Lepithiohydimerins A—D: Four pairs of neuroprotective thiohydantoin dimers bearing a disulfide bond from maca (*Lepidium meyenii* Walp.). *Chin. J. Chem.* **2021**, *39*, 2738-2744. doi:10.1002/cjoc.202100353
23. Tian, X.; Peng, X.; Yu, M.; Huang, Y.; Wang, X.; Zhou, L.; Qiu, M. Hydantoin and thioamide analogues from *Lepidium meyenii*. *Phytochem. Lett.* **2018**, *25*, 70-73. doi:10.1016/j.phytol.2018.03.011
24. Zhou, M.; Zhang, R.-Q.; Chen, Y.-J.; Liao, L.-M.; Sun, Y.-Q.; Ma, Z.-H.; Yang, Q.-F.; Li, P.; Ye, Y.-Q.; Hu, Q.-F. Three new pyrrole alkaloids from the roots of *Lepidium meyenii*. *Phytochem. Lett.* **2018**, *23*, 137-140. doi:10.1016/j.phytol.2017.12.002
25. Le, H.T.N.; Van Roy, E.; Dendooven, E.; Peeters, L.; Theunis, M.; Foubert, K.; Pieters, L.; Tuenter, E. Alkaloids from *Lepidium meyenii* (Maca), structural revision of macaridine and UPLC-MS/MS feature-based molecular networking. *Phytochemistry* **2021**, *190*, 112863. doi:10.1016/j.phytochem.2021.112863
26. Purnomo, K.A.; Korinek, M.; Tsai, Y.-H.; Hu, H.-C.; Wang, Y.-H.; Backlund, A.; Hwang, T.-L.; Chen, B.-H.; Wang, S.-W.; Wu, C.-C.; et al. Decoding multiple biofunctions of maca on its anti-allergic, anti-inflammatory, anti-thrombotic, and pro-angiogenic activities. *J. Agric. Food Chem.* **2021**, *69*, 11856-11866. doi:10.1021/acs.jafc.1c03485
27. Liu, J.H.; Zhang, R.R.; Peng, X.R.; Ding, Z.T.; Qiu, M.H. Lepipyrrolins A-B, two new dimeric pyrrole 2-carbaldehyde alkaloids from the tubers of *Lepidium meyenii*. *Bioorg. Chem.* **2021**, *112*, 104834. doi:10.1016/j.bioorg.2021.104834
28. Cui, B.; Zheng, B.L.; He, K.; Zheng, Q.Y. Imidazole alkaloids from *Lepidium meyenii*. *J. Nat. Prod.* **2003**, *66*, 1101-1103. doi:10.1021/np030031i
29. Jin, W.; Chen, X.; Dai, P.; Yu, L. Lepidiline C and D: Two new imidazole alkaloids from *Lepidium meyenii* Walpers (Brassicaceae) roots. *Phytochem. Lett.* **2016**, *17*, 158-161. doi:10.1016/j.phytol.2016.07.001
30. Le, N.T.; Foubert, K.; Theunis, M.; Naessens, T.; Bozdag, M.; Van Der Veken, P.; Pieters, L.; Tuenter, E. UPLC-TQD-MS/MS method validation for quality control of alkaloid content in *Lepidium meyenii* (Maca)-containing food and dietary supplements. *ACS Omega* **2024**, *9*, 15971-15981. doi:10.1021/acsomega.3c09356
